# A sensitive and robust analytical method for the determination of enramycin residues in swine tissues using UHPLC–MS/MS

**DOI:** 10.3389/fvets.2024.1462743

**Published:** 2024-09-03

**Authors:** Feifei Sun, Jicheng Qiu, Jingyuan Kong, Yuying Cao, Lin Li, Xingyuan Cao

**Affiliations:** ^1^Animal-Derived Food Safety Innovation Team, College of Animal Science and Technology, Anhui Agricultural University, Hefei, China; ^2^Department of Veterinary Pharmacology and Toxicology, China Agricultural University, Beijing, China; ^3^Key Laboratory of Detection for Veterinary Drug Residue and Illegal Additive, Ministry of Agriculture, Beijing, China

**Keywords:** enramycin, polypeptide antibiotic, residue, validation, UHPLC–MS/MS

## Abstract

Enramycin, a common growth promoter utilized in chickens and pigs, is sensitive against Gram-positive bacteria, and the maximum residue limit (MRL) of enramycin set up by is 30 μg/kg. However, the methods have been reported for detecting enramycin have failed to meet the accuracy requirements, with the required limit of quantification being higher than the MRL. To address this issue, we developed a high-sensitive and robust analytical method based on ultrahigh-performance liquid chromatography coupled with mass spectrometry (UHPLC–MS/MS), to determine enramycin residues in swine tissues, including liver, kidney, pork, and fat. The ENV cartridge was selected to cleanup and enrich analytes after being extracted using a mixture of 55% methanol containing 0.2 M hydrochloric acid. With comprehensively validation, this established method was found great linearity of enramycin in each tissue, with a coefficient of variation above 0.99. Satisfactory recoveries from four different spiking levels were acquired (70.99–101.40%) while the relative standard deviations were all below 9%. The limit of quantification of enramycin in the present study is 5 μg/kg in fat and 10 μg/kg in other tissues, meeting the requirements for conducting the corresponding safety evaluation study. This method was demonstrated with excellent specificity, stability, and high sensitivity. To conclude, this novel approach is sufficiently sensitive and robust for the safety evaluation of enramycin in food products.

## 1 Introduction

**Figure 1:**
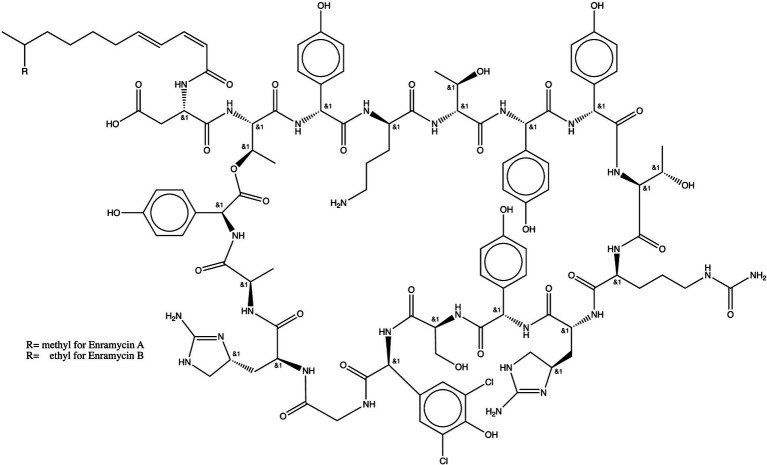
Chemical structure of enramycin (enramycin A and B).

Enramycin (also known as enduracidin), a 17-amino acid lipodepsipeptide, is consist of mixture of enramycin A and enramycin B (Figure 1) from the mycelium of soil bacterium Streptomyces fungicidicus No. B5477 Produced by Streptomyces fungicidicus against Alternaria solani (1, 2). Enramycin is used as a growth promoter on chickens and pigs, and has a strong antimicrobial activity against Gram-positive bacteria including vancomycin-resistant *enterococcus faecium* (VRE) and methicillin-resistant *staphylococcus aureus* (MRSA), and *N. gonorrhoeae* (2–5). Enramycin’s acting mechanism is by blocking the transglycosylation step of peptidoglycan biosynthesis (6). The action of mode for enramycin is analog to that of vancomycin, but they recognize different regions of transglycosylase substrate Lipid II (7, 8). The novel mechanism of action would contribute to a lack of cross-resistance with other antimicrobials, making enramycin a promising antimicrobial peptide for infections caused by Gram-positive bacteria. However, information on enramycin resistance with other human antimicrobials is not adequate. Investigations of acquiring antimicrobial-resistance have suggested possible generation of enramycin resistance in Gram-positive bacteria such as enterococcus and *Staphylococcus aureus* (9). However, there is no confirmatory method for the analysis of enramycin residue. It is, therefore, urgent to develop a robust method to monitor the appropriate use of enramycin.

Currently, there are limited methods available for accurately determining the presence of the polypeptide antibiotic enramycin in food-producing animals. One study which published in 1985 utilized a high-performance liquid chromatographic (HPLC) method to detect enramycin in swine and chicken muscles, which involved extraction with hydrochloric acid-acetone and liquid–liquid partition for clean-up. Enramycin was monitored at 230 nm by ultraviolet (UV) detector, and the limit of detection was 0.2 mg/kg for both muscles (10). Another study done by Inoue et al. utilized high-speed counter-current chromatography-tandem mass spectrometry equipped with electrospray in positive mode (HSCCC/ESI-MS) for the separation and determination of enramycin A and B, but the process was time-consuming and had insufficient sensitivity and accuracy for quantitation (11). A more recent study by Boison in 2015 developed a multi-residue method for determining seven polypeptide residues in chicken muscle by LC–MS/MS, which involved a two-step extraction and solid-phase extraction for cleanup before analysis. The limits of quantification for enramycin A and B were 66 μg/kg and 50 μg/kg, respectively (12). A highly sensitive and specific monoclonal antibody (mAb)-based indirect competitive enzyme-linked immunosorbent assay (ic-ELISA) was reported to detect enramycin residues in edible animal tissues in 2019, the limit of detection (LOD) were 144.8 μg/kg g and 98 μg/kg in the pork and chicken matrix (13). However, the MRL of enramycin as a growth promoter in chickens and pigs is 30 μg/kg for muscle, liver, kidney, and fat tissues in Japan and Korea. Therefore, there is a need to establish a sensitive analytical method to monitor enramycin for safety evaluation in food of animal origin.

Over recent years, UHPLC coupled to mass spectrometry has been a ubiquitous technique for accurately quantifying various compounds in a range of sample types (14–19). To address the limitations of previous methods, this study aimed to develop a sensitive, efficient, and validated analytical method for detecting enramycin A and B in swine tissues (pork, liver, kidney, fat) using UHPLC–MS/MS. The comprehensive approach involved method development, optimization, and validation, and the resulting data will aid in the further investigation of enramycin pharmacokinetics and residue depletion in swine.

## 2 Materials and methods

### 2.1 Chemicals and reagents

Enramycin A (purity, 95%) and enramycin B (purity, 85%) standards were procured from BOC Sciences (New York, United States). LC–MS grade methanol (MeOH), trifluoroacetic acid (TFA) and formic acid (FA) were purchased from Fisher Chemical Co. (New Jersey, United States), while HPLC grade ethyl acetate was obtained from the same supplier. Purified water was obtained via filtration using a Milli-Q system (Millipore, USA). All other chemicals and reagents, including analytical grade hydrochloric acid (HCl) and ammonium solution, were purchased from Sinopharm Chemical reagent Co., Ltd. (Shanghai, China). Bond Elut ENV cartridges (200 mg bed mass, 3 mL volume) were purchased from Agilent Technologies. C18 (500 mg bed mass, 6 mL volume), oasis HLB (500 mg bed mass, 6 mL volume), MCX (60 mg bed mass, 3 mL volume), WCX (60 mg bed mass, 3 mL) were purchased from Waters (Massachusetts, United States). Cleanert PCX (60 mg bed mass, 3 mL) was purchased from Troody Analytical Instrument Co., Ltd. (Shanghai, China).

### 2.2 Solutions preparation

The standard was dissolved into corresponding volume of methanol to prepare a final concentration of 1.0 mg/mL for enramycin A and B, respectively. A 100 μg/mL intermediate standard solution was obtained by transferring 1.0 mL of the stock solution into a 10 mL-volumetric flask and filling up with MeOH: 0.1 M HCl (8:2, v/v), followed by dilution into a series of standard working solutions of 20, 100, 125, 250, 500, and 1,000 ng/mL. The stock solutions and all the working solutions were stored at-20°C before analysis. 0.2 M HCl was prepared by pipetting 1.66 mL of concentrated HCl to a 100 mL volumetric flask, and bringing to volumetric line with purified water. MeOH (1.0% TFA) or water (1.0% TFA) was prepared by adding 1 mL TFA to a 100 mL volumetric flask filled with MeOH or water, correspondingly. 5% ammonia solution was prepared by adding 5.0 mL of ammonia to a 100 mL volumetric flask and filling up with water. Besides, other solutions were prepared in the same manner.

### 2.3 Preparation of standard calibration curves

A total of 100 μL of the prepared working solutions were diluted into 900 μL of blank matrix to prepare matrix matched standard curves (2, 10, 12.5, 25, 50, and 100 ng/mL). Besides, appropriate volumes of the pure standard enramycin A or B were peppited into neat solvent (MeOH: 0.1 M HCl, 8:2, v/v) to a final concentration of 2, 10, 12.5, 25, 50, and 100 ng/mL.

### 2.4 Sample extraction

A 2 g well-homogenized sample was accurately weighed into a 50 mL polypropylene tube. 10 mL 55% MeOH in purified water (55% MeOH-water) containing 0.2 M HCl was used to extract enramycin and the mixture was vortexed at 200 r/min for 10 min, followed by centrifugation at 10000 r/min for 10 min at 4°C. The supernatant was transferred into a new 50 mL polypropylene tube while the pellet was re-extracted twice using 10 mL of the same solution for each. The supernatant was combined, and the organic layer was evaporated to 12.5 mL. Then 2.5 mL of 0.2 M HCl was added to a total volume of 15 mL, followed by centrifugation at 4°C for 10 min at 10000 rpm before clean-up procedure.

### 2.5 Sample clean-up procedures

To purify the extracted sample, a Bond Elute ENV cartridge was used in the procedure. Prior to loading the extract, the cartridge was preconditioned sequentially with 5 mL of MeOH and 5 mL of water. The cartridge was then rinsed using 6 mL of MeOH: 5% ammonia (2:8, v/v) and dried under vacuum for 1 min. Subsequently, 6 mL of 1% ethyl acetate was loaded to rinse the cartridge, and vacuum was applied for 2 min to dry it. The target compound was then eluted using 8 mL of a mixture of MeOH and 0.2 M HCl (8,2, v/v) and vacuum dried for 1 min. The resulting mixture was filtered through a 0.22 μm microbore cellulose membrane into an auto-sampler vial for analysis using UHPLC–MS/MS.

### 2.6 Instrumental conditions

The quantitative determination of enramycin A and B were performed using the ultrahigh-performance liquid chromatography tandem Xevo TQS-micro (Waters, MA, United States). The chromatographic separations were achieved via ACQUITY UPLC BEH C18 column (2.1 × 50 nm, particle size 1.7 μm) using a gradient elution program at a flow rate of 0.3 mL/min. The mobile phase A was aqueous solution containing 0.2% FA while mobile phase B was MeOH. The gradient elution program was optimized as below: 0 min, 10% B; 1.5 min, 50% B; 3.3 min, 70% B; 3.5 min, 95% B; 6.0 min, 95% B; 6.1 min, 10% B; 9.0 min, 10% B. Besides, the injection volume was 10 μL. The data were acquired with a fitted an Agilent Jet Stream (AJS) electrospray ionization source in positive mode. The major parameters for mass spectrometer were: capillary voltage, 3.5 kV; dry gas temperature, 300°C; dry gas flow, 7 L/min; nebulier, 35 psi; sheath gas temperature, 300°C; sheath gas flow, 11 L/min; corona current, 0.16 μA. Multiple reaction monitoring (MRM) mode was used acquiring three product transition ions for every precursor ion. The transition ions including quantitative and confirmatory ions of enduracidin A and B were in detail described in Table 1.

**Table 1:** Detailed information on transition ions of enramycin including precursors and product ions in multiple reaction monitoring (MRM) mode.

Compound	Precursor ion	Retention time (min)	Transitions (*m/z*)	Collision energy (eV)
Enramycin A	[M + 3H]^3+^ *m/z* 786.1	2.4	786.1 > 1089.6	25
			786.1 > 178.9	25
			786.1 > 95.3	30
Enramycin B	[M + 3H]^3+^ *m/z* 790.9	2.6	790.9 > 1089.0	25
			790.9 > 193.2	25
			790.9 > 95.0	30

### 2.7 Method validation

The developed and optimized approach was comprehensively validated according to the guideline of European Medicines Agency (EMA) NO. 192217/2009, where the biological matrix effects, selectivity, calibration range and sensitivity, precision, accuracy, and stability were covered (20).

#### 2.7.1 Matrix effects

The matrix effects and selectivity are important parameters to evaluate the reliability and accuracy of the established analytical method. In this study, six blank matrix samples from different geographical regions were used to evaluate the matrix effects. The peak area of enramycin in the presence of matrix was compared to the peak area in the neat solvent to determine the degree of matrix effect.

Selectivity is the ability of an analytical method to distinguish the target analyte from other components in the sample matrix. In this study, the selectivity was investigated by analyzing six blank matrix samples from different sources for the estimation of potential interferences. The interfering components should be less than 20% of the lower limit of quantification for the target compounds, indicating that they would not interfere with the accurate quantitation of the target compounds.

#### 2.7.2 Carry-over

Estimating carry-over is an important parameter in method development to ensure that residual analyte from a high concentration sample does not contaminate subsequent blank samples. Carry-over is usually estimated by injecting a high concentration sample followed by blank samples and monitoring for any residual analyte signals. If the signal from the blank sample is within the acceptable range (usually less than 20% of the lower limit of quantification), then the carry-over is negligible.

#### 2.7.3 Accuracy and precision

The accuracy and precision of the method were evaluated based on the recoveries (R) and coefficient of variation (CV), respectively. The R and CV were calculated by spiking known amounts of the compound at three different concentration levels (LOQ, 0.5 MRL,1 MRL, 2 MRL). Each level was performed in six independent replicates, and the experiments were conducted for three consecutive days at each concentration level to evaluate the inter-day and intra-day precision as well as accuracy.

#### 2.7.4 Stability estimation

To carry out the stability studies, the low (15 μg/kg) and high (400 μg/kg) quality control (QC) samples were selected to ensure every procedure in the present study does not influence the accurate concentration of the analyte. QC samples were immediately prepared before analysis by spiking a certain amount of standard to blank matrix. Overall, the stability of the stock solution, freeze and thaw stability, short-term and long-term stability in matrix at room temperature, stability of processed samples at the auto-sampler.

## 3 Results and discussion

### 3.1 Instrumental conditions optimization

It has been reported that enramycin consists of enramycin A (molecular weight, MW, 2355.3) and enramycin B (MW, 2369.4) (11, 12). Standard solutions of enramycin A and enramycin B at the concentration of 500 ng/mL were infused into the electrospray ion source AJS positive (ESI^+^) and negative modes (ESI^−^) to optimize the mass spectrometric parameters. Enramycin A and B exhibited a superior response in ESI^+^. It is noteworthy that the polypeptides frequently generate status ions with double and triple charges (12, 21). The protonated ions in the triple-charge status of [(M + 3H) 3+] were produced in the present study by enramycin A and B, with mass to charge ratios (m/z) of 786.1 and 790.9, respectively. The top three intense fragment ions for enramycin A were m/z 1089.6, 178.9, and m/z 95.3, while the top three intense product ions for enramycin B were m/z 1089.0, 193.2, and m/z 95.0. These results were in line with other research (11, 21). Enramycin A and B were quantified using MRM mode, and the detailed qualitative and quantitative ions were shown in Table 1. Aside from that, the ACQUITY UPLC BEH C18 column (2.1 × 50 mm, particle size 1.7 μm) was used to obtain the chromatographic separations. The composition of mobile phase including acetonitrile, methanol, and formic acid, was evaluated. The results indicated that using MeOH and 0.2% FA as the mobile phases produced improved peak shapes and response intensities. The gradient elution program outperformed the isocratic elution program in removing the impact of impurities in the biological samples.

### 3.2 Sample preparation optimization

**Figure 2:**
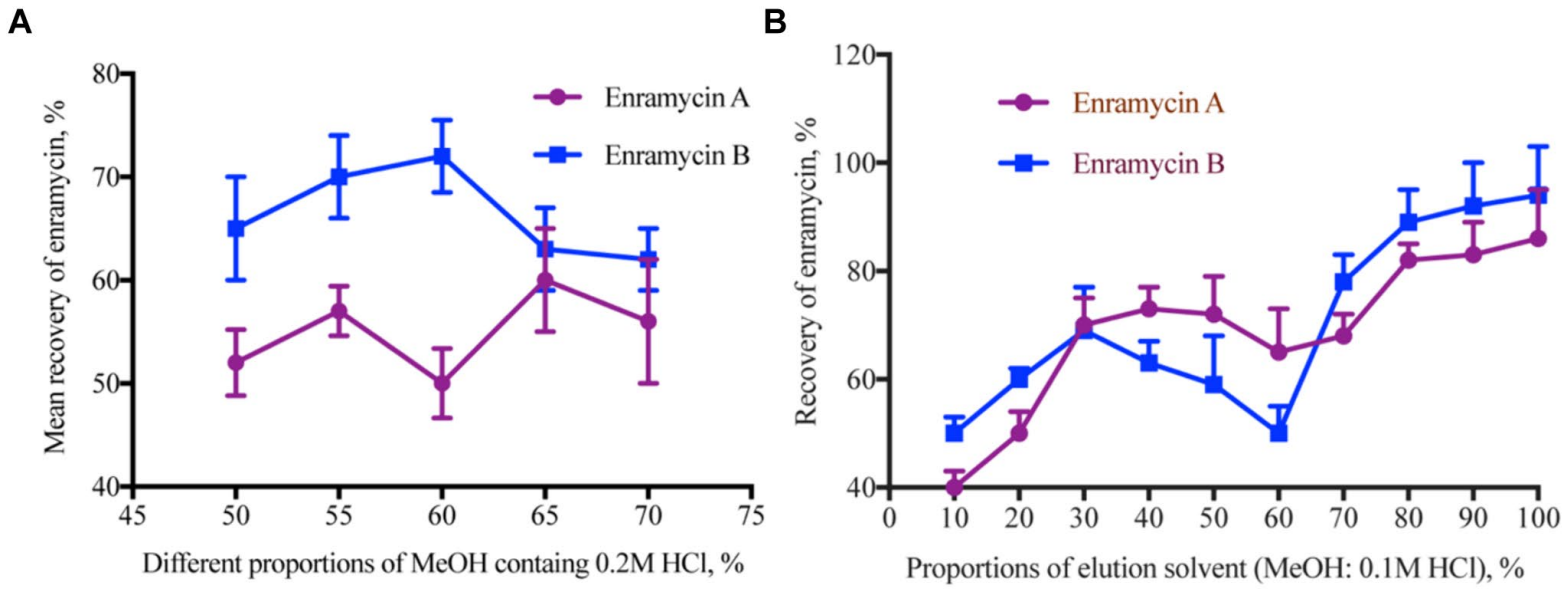
Optimization the extraction solution for enramycin from tissues. **(A)** Mean recoveries of enramycin using different proportions of MeOH containing 0.2 M HCl as the extracting solvent. **(B)** Mean recoveries of enramycin using different proportions of MeOH containing 0.1 M HCl as the elution solvent.

Accurate results of the study in the analysis of target chemicals from complex biological matrices depend on the selection of an adequate extraction solvent. A variety of solvents, including acetonitrile with 0.06% TFA and 1% TFA in an aqueous solution, as well as MeOH and MeOH with 1% TFA, were utilized as extraction solvents in previous research (10–12). Erythromycin consists of two amino groups that can react with HCl to generate the hydrochloride salt. This chemical process makes the erythromycin molecule more soluble in water and stable. The following extraction solvents were used in this investigation to assess the extraction efficiency: MeOH containing 2% FA, ethylenediaminetetraacetic acid (EDTA) phosphate buffer containing 2% TFA, a combination of MeOH and water (4:6, v/v), a combination of EDTA phosphate buffer and MeOH (4:6, v/v), a mixture of 1% TFA MeOH and 1% TFA (5:3, v/v), a mixture of 0.2 M HCl and MeOH (2:8, v/v), and MeOH containing 0.2 M HCl. The findings demonstrated that using 2% FA MeOH, a 0.2 M HCl and MeOH (2:8, v/v) combination, and 0.2 M HCl MeOH as the extraction solvent all led to better recoveries. Since enramycin dissolves in HCl, we also evaluated various MeOH concentrations (50, 55, 60, 65, and 70%) that included 0.2 M HCl. Enramycin A demonstrated the highest recoveries when 65% MeOH containing 0.2 M HCl was used, but enramycin B demonstrated the best recoveries when 60% MeOH containing 0.2 M HCl was used. By balancing the recoveries of enramycin A and B, peak shape, and peak purity, 55% MeOH containing 0.2 M HCl was chosen as the extraction solvent, as shown in Figure 2A. Two further extractions of the samples were necessary to achieve a acceptable recovery.

Furthermore, the organic solvents that had been acidified exhibited a potent precipitation capacity, which resulted in the disruption of natural components, particularly in the case of liver and kidney specimens. Therefore, extraction using 55% MeOH containing 0.2 M HCl was evaluated after the cleaning stage using solid phase extraction. To eliminate the unforeseen endogenous interferences, cartridges such as C18 (500 mg, 6 mL), oasis HLB (500 mg, 6 mL), MCX (60 mg, 3 mL), PCX (60 mg, 3 mL), and WCX (60 mg, 3 mL) were first calculated. Supplementary Table S1 of the supplemental materials contained detailed information on several cartridges. Nevertheless, enramycin A and B recoveries were both less than 50%, which may have been brought on by the cartridges’ tiny pores. For the subsequent purification, a cartridge with a higher pore size was selected due to the large molecular weight, ENV (200 mg, 3 mL). The contaminant in liver tissue may be rinsed off using a 5% ammonia solution and MeOH, according to results obtained using MCX cartridges. Since there were no target chemicals in the washing solution, the 80% MeOH 5% ammonia solution proved to be the best choice for the current investigation. Besides that, 1%FA-containing ethyl acetate was utilized later to eliminate the lipid interferences in the matrix. By varying the ratios of 0.1 M HCl and MeOH (10, 20, 30, 40, 50, 60, 70, 80, 90, 100%), the elution solvent was assessed. Excellent recoveries were obtained when the elution solvents were 80–100% MeOH. When 90 and 100% MeOH were used to elute the cartridge, more interferences were noticed in the chromatogram. As a result, 8 mL of a 0.2 M HCl and MeOH (2:8, v/v) combination was chosen as the elution solvent, and Figure 2B illustrates the acceptable recovery.

### 3.3 Method validation

**Figure 3:**
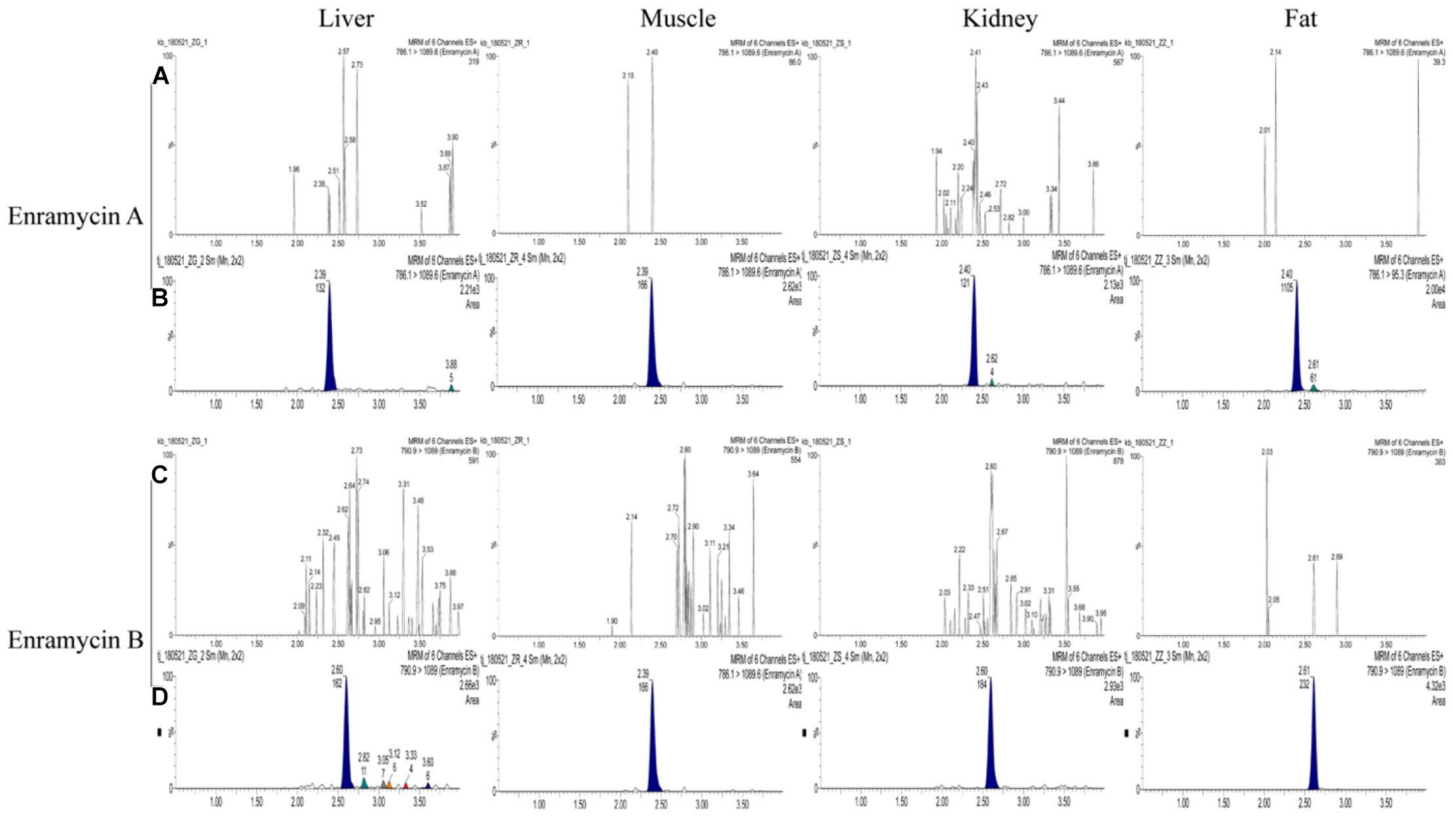
Chromatograms of enramycin A and enramycin B in swine tissues. **(A,C)** Chromatograms of blank tissue samples; **(B)** Chromatograms of blank samples fortified with enramycin A at LOQ (limit of quantification) level, where 786.1 > 1089.6 was the quantitative ions; **(D)** Chromatograms of blank samples fortified with enramycin B at LOQ (limit of quantification) level, where790.9 > 1,089 was the quantitative ions.

In terms of matrix effects, selectivity, calibration range and sensitivity, precision, accuracy, and stability, the devised approach was validated. In the six blank biological samples of each tissue, there was not an interfering peak within the time duration of target peaks, suggesting the method’s selectivity. The standard enramycin chromatograms in the blank tissue samples are shown in Figure 3A, along with additional data in Supplementary Figure S1. The matrix effects investigation confirmed that the signals for enramycin A and B were significantly inhibited in the tissues of pigs and chickens. For the subsequent quantification, matrix-matched standard curves with acceptable linearity were employed (Table 2).

**Table 2:** Matrix-matched standard curves of enramycin in swine tissues.

Compound	Tissue	Equation	Linearity range (μg/kg)	Correlation coefficient (r^2^)
Enramycin A	Pork	y = 706.83x-700.68	2 ~ 100	0.9997
	Kidney	y = 829.52x-746.96	2 ~ 100	0.9990
	Liver	y = 614.26–347.36	2 ~ 100	0.9985
	Fat	y = 1005.01x-3211.35	2 ~ 100	0.9997
Enramycin B	Pork	y = 319.27x-91.33	2 ~ 100	0.9993
	Kidney	y = 57.95x + 89.66	2 ~ 100	0.9989
	Liver	y = 206.21x + 424.30	2 ~ 100	0.9995
	Fat	y = 1035.20x-742.5	2 ~ 100	0.9992

Less than 20% of the LOQ was carried over. The lowest concentration of the target molecule in this investigation that could be precisely and satisfactorily measured was known as the LOQ. The lowest calibration point was thought to be the LOQ. Furthermore, the LOQ exceeded a blank sample’s signal by five times. As three times the signal of a blank sample, the LOD was defined. The calibration standards that were lowest and highest, respectively, were LOQ and ULOQ. For LOQ, each calibration standard’s recovery satisfied the requirements within 100 ± 15% or 100 ± 20% for LOQ.

**Figure 4:**
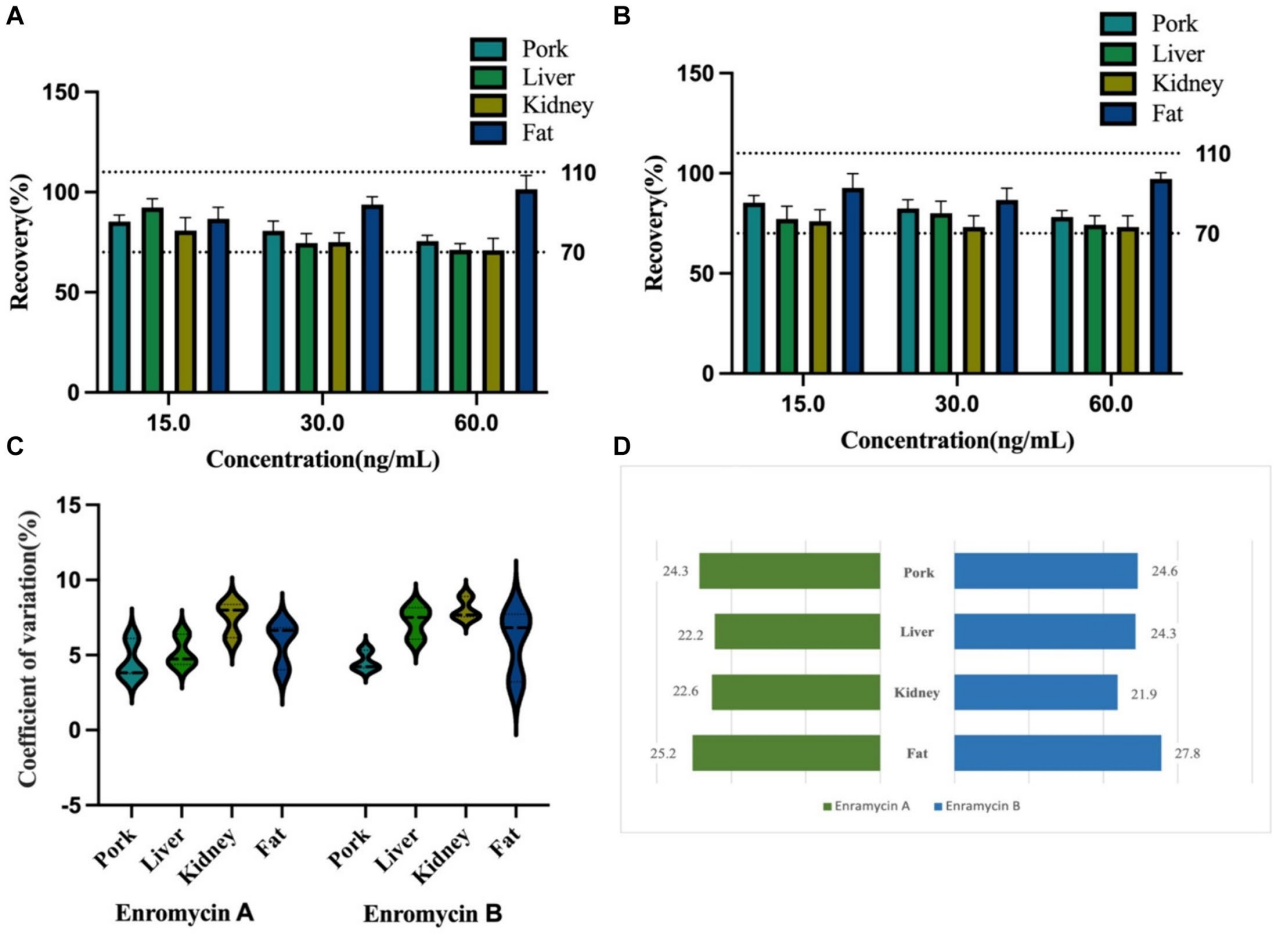
The precision and accuracy validation of enramycin in various tissues of swine, and detection results of simulated samples. **(A)** The detailed information on accuracy of enramycin A in swine tissues; **(B)** The detailed information on accuracy of enramycin A in swine tissues; **(C)** The detailed information on accuracy of enramycin A in swine tissues; **(D)** Samples were spiked with enramycin at a concentration of 25 μg/kg to simulate incurred samples.

Compared to other published techniques, the LOQ for enramycin A and B established in this study were more sensitive, measuring 5 μg/kg in fat and 10 g/kg in tissues, and both the LOD for enramycin A and B established in this study were 3 μg/kg in tissues (10–12). Moreover, the typical chromatograms of enramycin in the blank tissue samples spiked with enramycin A and B at LOQ level were shown in Figure 3B. Since the MRLs (30 μg/kg) of enramycin in chicken and swine were established by Japan and Korea, the established method met the requirements for regulatory use. Furthermore, three different concentrations at LOQ (5 μg/kg or 10 μg/kg), 0.5 MRL (15 μg/kg), 1 MRL (30 μg/kg), 2 MRL (60 μg/kg) were selected to evaluate the accuracy and precision. Coefficient of variation (CV, %) and recoveries were used to evaluate accuracy and precision. The mean recoveries of enramycin A and B at three distinct concentration levels in each tissue were within the range of 70.99 to 101.40%, which were within the permissible limit defined by European Union regulation. The results showed that the intra-day and inter-day precision was all below 9%. Figures 4A–C provided a full description of the accuracy and precision data. The stability results showed that after 3 months of storage at either-20°C or 4°C, enramycin A and B remained stable. It is best to examine biological samples within the minimum freeze–thaw cycle because it has been discovered that enramycin degrades after a freeze–thaw cycle. Furthermore, enramycin, the analyte, remained constant in the tissues during the extraction and cleansing procedures in the auto-sampler. This suggests that enramycin is stable in the tissue matrix and continues to be stable in the auto-sampler, which was kept at 4°C. Decrease the freeze–thaw cycle when detecting the samples, as this is crucial. Furthermore, the comprehensive stability assessment findings could be found in the supplemental data (Supplementary Table S2).

### 3.4 Application

In order to simulate the incurred samples in the absence of incurred samples, enramycin was added to the blank samples at a dose of 25 μg/kg. The findings shown in Figure 4D proved that the technique may be used to precisely identify and measure enramycin in swine tissues. The created and verified approach yielded data that was not significantly different from the known quantity, indicating the method’s dependability in real-world applications. The applicability of this approach for the detection of enramycin in chicken liver, kidney, muscle, and fat has also been verified (unpublished data).

## 4 Conclusion

A highly sensitive and reliable analytical method based on UHPLC–MS/MS was developed for the determination of enramycin (A, B) in swine tissues including liver, kidney, muscle, and fat. This method underwent comprehensive validation, covering selectivity, sensitivity, standard curves, accuracy, precision, and stability. Excellent validation characters were obtained. The LOQ was 5 μg/kg in fat and 10 μg/kg in other tissues, which was more sensitive than previously published methods, and lower than MRL of enramycin in tissues (30 μg/kg). This is the first method available meeting the fit for purpose criteria. In conclusion, our method has been proven to be accurate and robust for monitoring enramycin residues level in different tissues, further facilitating its safety evaluation in food products.

## Data Availability

The original contributions presented in the study are included in the article/Supplementary material, further inquiries can be directed to the corresponding author/s.
