# Supplementary material for: A sensitive and robust analytical method for the determination of enramycin residues in swine tissues using UHPLC–MS/MS
Source: Front Vet Sci. 2024 Sep 3;11:1462743. doi: 10.3389/fvets.2024.1462743 (PMC11405371; doi:10.3389/fvets.2024.1462743)
Supplement: Supplementary file 1 [file Data_Sheet_1.docx]

**Supplementary**

**Supplementary Tables**

Table S1 Specific information on different cartridges

Table S 2 Stability evaluation

**Supplementary Figure**

Figure S1 Chromatograms of blank sample fortified with enramycin A or B at LOQ (limit of quantification) level (15 μg/kg), where three pairs of transition ions were covered. 790.9>1089 and 786.1>1089.6 were the quantitative ions for enramycin A and B, respectively, while the other transition ions were selected as the qualitative ions.

Table A.1 Specific information on different cartridges

| Cartridge | Activation solvents | Rinse solutions | Elution solvents |
| --- | --- | --- | --- |
| C18  (500 mg, 6mL) | 6 mL MeOH, 6mL water | 4 mL AA containing 1%FA;  4mL n-hexane | 3 mL MeOH |
| HLB  (500 mg, 6mL) | 6 mL MeOH, 6mL water | 4 mL AA containing 1%FA;  4mL n-hexane | 3 mL MeOH |
| MCX  (500 mg, 3mL) | 3 mL MeOH, 3mL water | 4 mL aqueous solution containing 2%FA;  4mL MeOH | 6 mL MeOH containing 5% ammonia |
| PCX  (500 mg, 3mL) | 3 mL MeOH, 3mL water | 4 mL aqueous solution containing 2%FA;  4mL MeOH | 6 mL MeOH containing 5% ammonia |
| WCX  (500 mg, 3mL) | 3 mL MeOH, 3mL water | 4 mL aqueous solution containing 5% ammonia;  4mL MeOH | 6 mL MeOH containing 2% FA |

Where, MeOH, methanol; AA, acetic acetate; FA, formic acid.

Table A. 2 Stability evaluation

|  | Spiking Conc. (μg/kg) | Coefficient of variation (%) | |
| --- | --- | --- | --- |
|  |  | Enramycin A | Enramycin B |
| Short-term stability | 15 | 9.8 | 11.4 |
|  | 400 | 8.3 | 9.5 |
| Long term temperature | 15 | 12.5 | 11.9 |
|  | 400 | 12.1 | 10.8 |
| Stock solution | 15 | 8.7 | 8.2 |
|  | 400 | 9.8 | 10.3 |
| Freeze-thaw stability | 15 | 18.1 | 20.5 |
|  | 400 | 19.8 | 21.7 |
| Stability in autosamper | 15 | 9.1 | 10.9 |
|  | 400 | 7.9 | 8.7 |


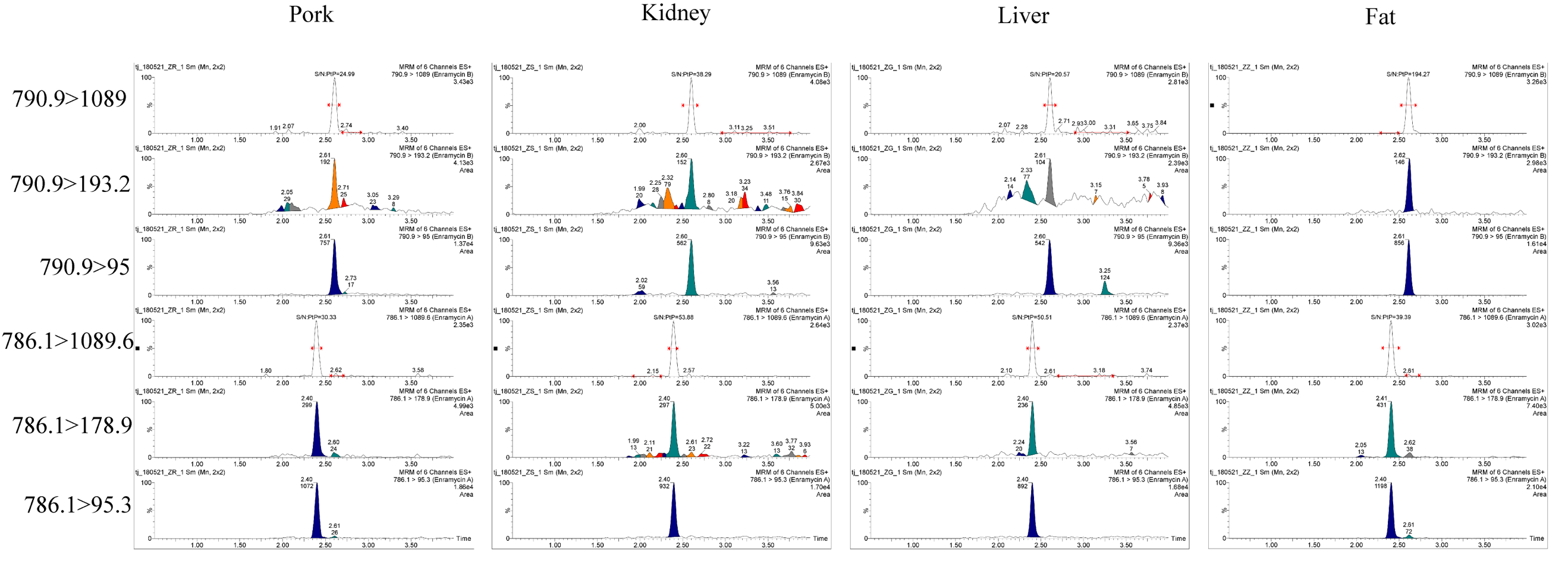


Figure S1 Chromatograms of blank sample fortified with enramycin A or B at LOQ (limit of quantification) level, where three pairs of transition ions were covered. 790.9>1089 and 786.1>1089.6 were the quantitative ions for enramycin A and B, respectively, while the other transition ions were selected as the qualitative ions.
